# Blast Injury in the Spine: Dynamic Response Index Is Not an Appropriate Model for Predicting Injury

**DOI:** 10.1007/s11999-015-4281-2

**Published:** 2015-04-01

**Authors:** Edward Spurrier, James A. G. Singleton, Spyros Masouros, Iain Gibb, Jon Clasper

**Affiliations:** Royal Centre for Defence Medicine, Birmingham, UK; Department of Bioengineering, Imperial College London, London, SW7 2AZ UK

## Abstract

**Background:**

Improvised explosive devices are a common feature of recent asymmetric conflicts and there is a persistent landmine threat to military and humanitarian personnel. Assessment of injury risk to the spine in vehicles subjected to explosions was conducted using a standardized model, the Dynamic Response Index (DRI). However, the DRI was intended for evaluating aircraft ejection seats and has not been validated in blast conditions.

**Questions/purposes:**

We asked whether the injury patterns seen in blast are similar to those in aircraft ejection and therefore whether a single injury prediction model can be used for both situations.

**Methods:**

UK military victims of mounted blast (seated in a vehicle) were identified from the Joint Theatre Trauma Registry. Each had their initial CT scans reviewed to identify spinal fractures. A literature search identified a comparison population of ejected aircrew with spinal fractures. Seventy-eight blast victims were identified with 294 fractures. One hundred eighty-nine patients who had sustained aircraft ejection were identified with 258 fractures. The Kruskal-Wallis test was used to compare the population injury distributions and Fisher’s exact test was used to assess differences at each spinal level.

**Results:**

The distribution of injuries between blast and ejection was not similar. In the cervical spine, the relative risk of injury was 11.5 times higher in blast; in the lumbar spine the relative risk was 2.9 times higher in blast. In the thoracic spine, the relative risk was identical in blast and ejection. At most individual vertebral levels including the upper thoracic spine, there was a higher risk of injury in the blast population, but the opposite was true between T7 and T12, where the risk was higher in aircraft ejection.

**Conclusions:**

The patterns of injury in blast and aircraft are different, suggesting that the two are mechanistically dissimilar. At most vertebral levels there is a higher relative risk of fracture in the blast population, but at the apex of the thoracic spine and in the lower thoracic spine, there is a higher risk in ejection victims. The differences in relative risk at different levels, and the resulting overall different injury patterns, suggest that a single model cannot be used to predict the risk of injury in ejection and blast.

**Clinical Relevance:**

A new model needs to be developed to aid in the design of mine-protected vehicles for future conflicts.

## Introduction

Improvised explosive devices (IEDs) have featured prominently in recent insurgent warfare and are used in attacks outside the context of warfare worldwide [23]. A large number of landmines remain in historical battlefields with a huge effort to clear them and a persistent risk to the civilian population and humanitarian aid workers. The global military population is also continuously engaged in operations with a risk of attack by IEDs. There will be, therefore, a need to protect personnel from such attacks for many years.

**Fig. 1:**
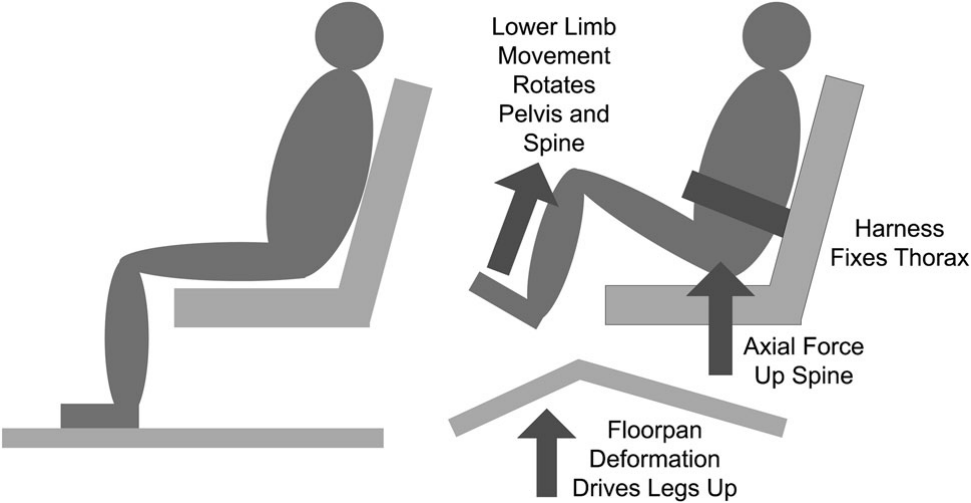
The effect of underbody blast on a seated victim: the blast beneath the vehicle drives the seat up and deforms the floor, transferring force to the spine through the pelvis and lower limbs.

When a buried IED detonates under a vehicle (underbody blast), a supersonic shockwave forms, carrying a mass of ejected soil toward the underside of the vehicle and imparting a large force because the pressure wave may reach 3 million psi [3, 8, 23]. The magnitude of this imparted force is difficult to quantify because it depends on the size of the device, which is not often known in the case of an insurgent attack. This deforms the vehicle floor and accelerates the whole vehicle upward. The vehicle occupants are therefore subjected to high accelerations that are primarily vertical and may lead to lower extremity, pelvis, and spinal fractures [23]. In addition to the axial injury mechanism to the spine, the floorpan deforms, which may drive the legs up, rotating the pelvis and flexing the lumbar spine, thus affecting the loading of individual vertebrae and the pattern of resulting fractures (Fig. 1) [15, 22].

National authorities use standardized tests mandated by the North Atlantic Treaty Organisation (NATO) to assess vehicles and their modifications such as energy-dissipating seats under underbody blast attack conditions [19]. The risk of spinal injury is assessed based on the movement of a test dummy’s pelvis and is calculated using the Dynamic Response Index (DRI), which was originally developed to aid in the design of aircraft ejection seats [2, 9, 10]. However, although ejection seats use explosive charges and rocket systems to accelerate rapidly vertically and are associated with a high risk of spinal injury, the forces involved are an order of magnitude lower than those associated with blast injury with peak acceleration from ejection seats approximately 20 G and acceleration in blast incidents of over 100 G [1, 26]. DRI is a probabilistic model and does not itself attempt to predict injury patterns; however, if the model is to be valid in both blast and aircraft ejection scenarios, the mechanism and therefore pattern of injury must be similar. The data to calculate DRI for historical blast incidents are not available, so a direct comparison and validation is not possible; DRI must therefore be validated or refuted in blast indirectly. DRI has not been validated in blast, so its ability to predict injury in the underbody blast environment is actually unknown. The DRI model has been validated for ejection injury with ballistic seat designs, although it does appear to underestimate the risk of injury with rocket-assisted seat systems [2].

We therefore asked whether the injury patterns seen in blast are similar to those in aircraft ejection and, therefore, whether a single injury prediction model can be used for both situations. We theorized that if the injury patterns in blast are similar to those seen in ejection, then a single simple model such as the DRI might be applicable to both scenarios, but if the injury patterns are different, then the two situations are sufficiently disparate that separate models are needed.

## Materials and Methods

We sought to compare the injury patterns between a group of ejection injury victims and a group of IED blast victims. A group of victims of underbody blast was identified from the UK military population. A second group, consisting of ejected aircrew with spinal fractures, was identified from published literature.

The United Kingdom Ministry of Defence maintains a database of all wounded personnel, listing their injuries and the circumstances in which those injuries occurred. Victims of blast injury were identified by searching the Joint Theatre Trauma Registry (JTTR) database [24], with the consent of the Royal Centre for Defence Medicine, for patients with spinal fractures who had been exposed to blast in an IED strike against a vehicle. Survivors and fatalities were included. Having identified the relevant casualties, we analyzed the relevant imaging. Surviving patients had their initial CT scan, performed during emergency department resuscitation, reviewed to identify vertebral fractures. With the consent of the coroner, fatal victims had their CT postmortem images reviewed to identify vertebral fractures by the lead author (ES). Where there was doubt, a consultant radiologist (IG) confirmed the correct fracture classification. The fractured vertebrae in each case were recorded in a simple database.

**Fig. 2:**
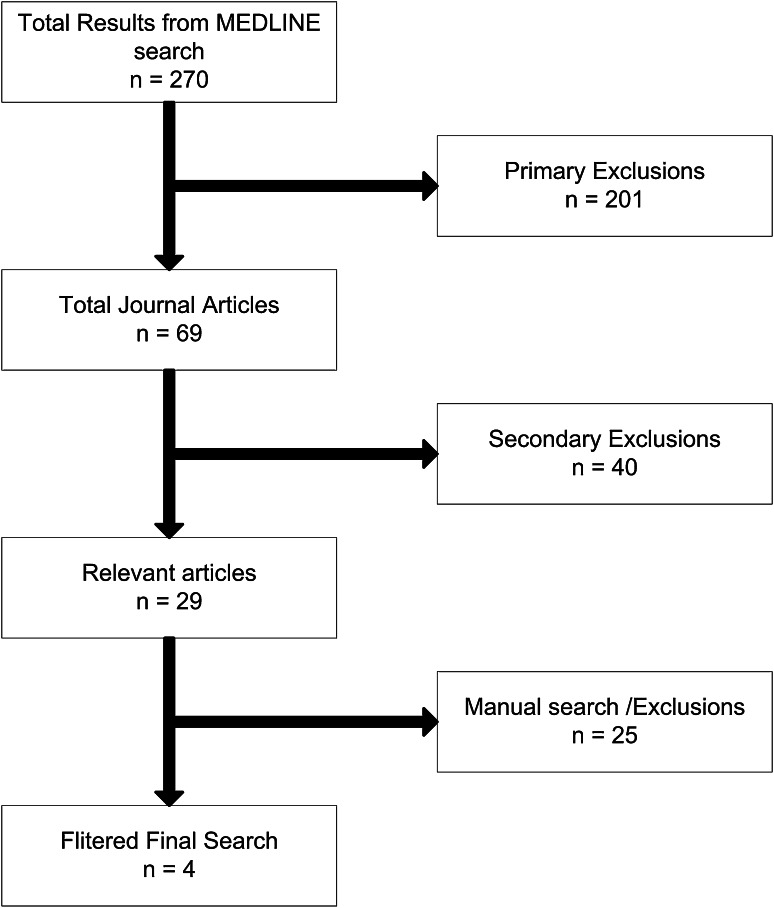
Flowchart showing the literature search on ejection injuries of the spine.

A literature review was carried out to identify the vertebral fracture distribution for aircraft ejection in the published literature (Fig. 2). A MEDLINE^®^ search using the terms “aircraft ejection spine”, “ejection spine”, and “aircraft ejection” was performed and each paper was reviewed for references to expand the search. Primary exclusions were made for papers that did not relate to aircraft ejection, were not in English, or were not available by the lead author (ES). Secondary exclusions were made if it was not possible to identify the individual vertebral levels injured or to identify which patients did and did not have fractures at any given level. Four papers were identified with sufficient detail for the ejection cohort of this study. They reported 258 fractures in 189 patients [13, 16, 17, 20].

Seventy-eight victims of blast injury with spinal fractures were identified in the JTTR search. Of those, 53 were survivors and 25 were fatalities. The mean age was 26 years (range, 18–55 years). There were 294 vertebral fractures in these 78 patients with a mean of 3.8 per patient (range, 1–22).

We theorized that the patterns of injury in these two groups may be different. To identify any difference in the injury patterns between these two groups, the Kruskal-Wallis test was used to compare the distribution between the number of fractures at each level. Contingency tables were constructed for the cervical, thoracic, and lumbar spinal regions and for each vertebral level, and Fisher’s exact test was used for post hoc analysis to identify a statistically significant difference in the risk of vertebral injury at each level and region. Relative risk was calculated to measure effect size and direction. Statistical analysis was performed using SPSS (IBM, Chicago, IL, USA). A significance level of p < 0.05 was defined for all tests.

## Results

**Fig. 3:**
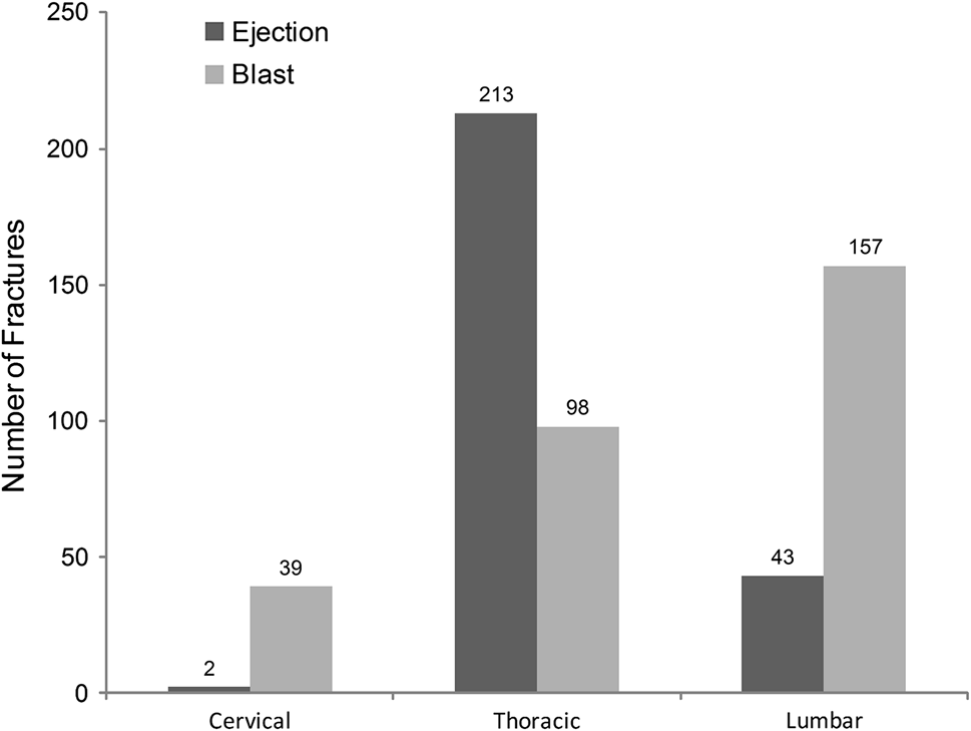
Distribution of injuries in the blast and ejection victim cohorts is shown.

In the blast injury group, the most common thoracolumbar fracture patterns were wedge compression (n = 44) and burst (n = 50) (Fig. 3). For the ejection injury group, the literature used for this study did not classify the fractures anatomically or mechanistically. Analysis of the distribution of injury between the two groups with the Kruskal-Wallis test showed no difference in the overall distribution (p *=* 0.317). However, analysis by region (Table 1) suggests differences in the cervical and lumbar regions (p = 0.001) but not in the thoracic region (p = 1.00). Further examination at the level of individual vertebrae (Tables 2, 3) supports the likelihood of a fracture at a given level is not similar for blast and ejection victims, particularly in the cervical and lumbar regions. At all lumbar and cervical levels, there is a higher relative risk of fracture in the blast population. There is a higher risk of fracture in blast in the upper thoracic spine, but from T7 to T12, the risk is higher in ejection.

**Table 1:** Number of patients with fractures in each spinal region with p value by Fisher’s exact test*

Region	Ejection, number (%)	Blast, number (%)	p value	Relative risk blast/ejection	95% CI of RR
Cervical	4 (2)	19 (24)	0.001	11.5	4.1–32.7
Thoracic	93 (49)	39 (50)	1.000	1.0	0.8–1.3
Lumbar	24 (13)	54 (69)	0.001	2.9	3.6–8.1

* p < 0.05 suggests that a significant difference exists; CI = confidence interval; RR = relative risk.

**Table 2:** Number of patients with fracture at each vertebral level with p values by Fisher’s test*

Vertebra	Ejection, number (%)	Blast, number (%)	p value	Relative risk blast/ejection	95% CI of RR
C1	0 (0)	5 (6)	0.0019	N/A	
C2	0 (0)	6 (8)	< 0.001	N/A	
C3	0 (0)	4 (5)	0.0069	N/A	
C4	0 (0)	3 (4)	0.0243	N/A	
C5	0 (0)	2 (3)	0.0846	N/A	
C6	2 (1)	8 (10)	0.0011	9.69	2.1–44.6
C7	0 (0)	11 (14)	0.001	N/A	
T1	0 (0)	6 (8)	< 0.001	N/A	
T2	0 (0)	4 (5)	0.0069	N/A	
T3	2 (1)	4 (5)	0.0622	4.85	0.9–25.9
T4	7 (4)	9 (12)	0.0216	3.12	1.2–8.1
T5	12 (6)	12 (15)	0.0316	2.42	1.1–5.2
T6	21 (11)	12 (15)	0.4132	1.38	1.1–2.7
T7	24 (13)	8 (10)	0.6811	0.81	0.4–1.3
T8	28 (15)	7 (9)	0.2353	0.61	0.3–1.3
T9	27 (14)	10 (13)	0.8470	0.90	0.5–1.8
T10	22 (12)	6 (8)	0.3881	0.66	0.3–1.2
T11	30 (16)	7 (9)	0.1735	0.57	0.3–1.4
T12	40 (21)	13 (17)	0.5002	0.79	0.5–1.4
L1	29 (15)	33 (42)	0.001	2.76	1.8–4.2
L2	7 (4)	28 (36)	0.001	9.69	4.4–21.3
L3	4 (2)	28 (36)	0.001	16.96	6.2–46.8
L4	2 (1)	23 (29)	0.001	27.87	6.7–115.4
L5	1 (1)	17 (22)	0.001	41.19	5.6–304.2

* p < 0.05 suggests that a significant difference exists; CI = confidence interval; RR = relative risk; N/A = cannot calculate as none in the ejection group.

**Table 3:** Number of victims with fractures in each group*

Vertebra	Ejection	Blast	p value
C1	0	5	0.0019
C2	0	6	< 0.001
C3	0	4	0.0069
C4	0	3	0.0243
C5	0	2	0.0846
C6	2	8	0.0011
C7	0	11	< 0.001
T1	0	6	< 0.001
T2	0	4	0.0069
T3	2	4	0.0622
T4	7	9	0.0216
T5	12	12	0.0316
T6	21	12	0.4132
T7	24	8	0.6811
T8	28	7	0.2353
T9	27	10	0.8470
T10	22	6	0.3881
T11	30	7	0.1735
T12	40	13	0.5002
L1	29	33	< 0.001
L2	7	28	< 0.001
L3	4	28	< 0.001
L4	2	23	< 0.001
L5	1	17	< 0.001

* Significance by Fisher’s exact test.

## Discussion

The IED is often encountered in current conflicts, and landmines are a hazard of both current and historical wars. When one of these devices detonates beneath a vehicle, it causes devastating injury to the occupants with a high risk of spinal injury. Reducing the risk of spinal injury is of interest to vehicle designers, but no specifically validated model exists to help test design features. At present, the industry uses the DRI, initially developed to test ejection seats but adopted for blast tests without good evidence of its applicability to the blast scenario. This article sought to test whether DRI should be used for the two separate situations on the premise that DRI would be valid for both if the pattern, and therefore mechanism, of injury is similar. It is clear that the patterns of injury are different, so the mechanisms of injury are dissimilar and DRI should therefore not be used for both blast and ejection.

Accepting that DRI has been validated in ejection seat injury, this article sought to validate DRI by comparing the injury patterns between blast and ejection [2]. There are limitations to this approach. This article compared the published literature with respect to aircraft ejection injuries with a study population of blast victims. The papers describing injury patterns in ejection provide limited detail, and the usable sample of the total population of ejected aircrew was small. However, we used the whole UK population of mounted blast victims with spinal fractures as a comparison population. The small populations in both groups of this study were therefore a limitation demonstrated by the wide confidence intervals of relative risk. Additionally, the design of this study cannot alone refute the utility of DRI in blast injuries; it can only show that the injury patterns in blast and ejection are different, which suggests that different prediction models are necessary for each mechanism of injury. As a simple probabilistic model that aims only to estimate the risk of fracture in a given scenario, DRI does not attempt to predict injury patterns; it simply estimates the risk of injury based on a given pelvic and spinal displacement after blast. It would be interesting to analyze the DRI prediction of spinal injury in the incidents that lead to the injuries described in this article, and this would add useful data to the validation or refutation of the DRI model in blast. However, the displacement and acceleration of the vehicle floor and seat are not known in real-world incidents so it is not possible to calculate DRI for historical incidents. It would be better to validate DRI by direct means such as this, but the limited information available means that indirect means of validation must be sought.

This article aimed to identify whether the DRI model is clinically valid for predicting spinal injury in underbody blast attacks against vehicles. However, the behavior of the spine is complex, and the behavior of all its elements at the high loading rates seen in blast is not well understood. The validity of a model like DRI depends on the behavior of the model in vitro matching the behavior of the spine in vivo [21]. Not enough is known about the behavior of the spine in blast to validate DRI in this manner. The DRI model may be as valid in blast as it is in ejection if the patterns of injury in each group are demonstrably similar. In this study, the ejection injury group had more thoracic than lumbar or cervical spine injuries. The blast group had a higher incidence of lumbar fractures. This analysis shows that there is a different risk of fracture at all levels of the lumbar spine and at all but one level of the cervical spine. There was no difference in the risk of fracture in the lower thoracic spine, where the majority of ejection seat victims were injured. The direction of the difference varies at different levels of the spine with a higher risk of fracture in blast at most levels but a higher risk in ejection from T7 to T12, which further supports the notion that the mechanism of injury must be different. Therefore, given that the injury pattern is very different, the mechanism of injury is likely different between the two groups and therefore DRI should not be used in both blast and ejection injury prediction.

DRI was developed by Latham and described by Stech and Payne [25]. The model describes the spine as a simple spring and damper system, supporting the mass of the torso above the pelvis. In the NATO standard blast tests, where a specified charge is detonated beneath the vehicle, the DRI is calculated using the pelvic displacement of a standard anatomical test device (ATD), the Hybrid III (Humanetics, Plymouth, MI, USA), and is used to predict the risk of spinal fracture [18, 19]. The Hybrid III has a rigid lumbar spine. This is a critical limitation because the effect of the dummy’s torso mass on pelvis movement is very different from that seen in humans. DRI then estimates the behavior of the spine based on a simple single spring-damper system when in reality the spine is a complex system of vertebrae linked by discs, ligaments, and muscles that changes its characteristics as it moves [27]. The model is designed for pure axial loads; if there is a change in the force vector or spinal alignment, DRI has no way to correct for it. The limitations of DRI suggest that it is unlikely to give useful data in complex situations; this study shows that ejection and blast injury have different mechanisms and therefore that DRI should not be used for both scenarios. An improved model is therefore needed, perhaps one that allows for changes in the position of the spine and direction of the force during a blast or ejection event. There have been several attempts to improve DRI. Chandler [6] correlated DRI with a peak compression force exerted on the ATD spine to indicate spinal fracture risk. A further development of DRI for vertical impact tests is the Spinal Injury Criterion [11]. Neither of these has been proven to be better than DRI.

The current spinal injury prediction models therefore share common limitations: they are based on a very simple model of the behavior of the spinal column under blast loading and are designed to be applied to a test dummy that was not intended for vertical loading tests. Yoganandan et al. [28] recently examined the thoracolumbar spine in high-rate axial loading, producing an injury tolerance curve based on the peak axial force. However, this experiment used a small number of specimens in a fixed posture. In underbody blast, passengers in different positions can be expected to be in different postures, so a risk prediction model needs to incorporate the effect of posture and movement during the blast event. Current research is divided into several strands. Basic research is evaluating the behavior of ligaments and vertebral bodies at high strain rates similar to those seen in blast [4, 5, 10]. These can be correlated with work on the injury patterns seen in blast to attempt to derive the posture of the spine at the moment of failure and to postulate the mechanism of failure [7, 12, 22]. For example, Lehman et al. [15] suggest that in underbody blast, the use of body armor by soldiers reduces the mobility of the upper lumbar spine and increases the risk of low lumbar burst fractures. Ragel et al. [22] suggest that the seat harness provides a fulcrum about which the lumbar spine rotates, producing flexion-distraction injuries in the lumbar vertebra. Each of these strands improves understanding of the behaviour of parts of the lumbar spine that can then be brought together to produce a simulation of the whole system’s behavior in blast. Improvements in computer power over recent years have allowed development of simulations of the whole spine [14]. Between computational simulation and cadaveric tests, it is hoped that a reliable metric may be produced to correlate vehicle movement or dummy response with the risk of spinal injury and therefore accurately assess the risk of injury in blast tests and help improve vehicle design in the future.

The DRI is a simple model of the spine that does not accurately reflect the behavior of the spine under blast loads. When coupled with a test dummy that was not intended to be used for underbody blast tests, the data from underbody blast experiments probably do not give a true indication of the risk of spinal fracture. We found that the conjecture that a model suitable for predicting injury risk in aircraft ejection seats would also be satisfactory in blast is not correct. A multistranded approach to spinal injury prediction research is therefore needed, where the mechanism of injury in blast is properly understood, the behavior of the spine under blast loads established, and a risk prediction model developed based on detailed understanding of this and the material properties of the spinal column.
